# “Everything You Always Wanted to Know about Sex (but Were Afraid to Ask)” in *Leishmania* after Two Decades of Laboratory and Field Analyses

**DOI:** 10.1371/journal.ppat.1001004

**Published:** 2010-08-19

**Authors:** Virginie Rougeron, Thierry De Meeûs, Sandrine Kako Ouraga, Mallorie Hide, Anne-Laure Bañuls

**Affiliations:** 1 Génétique et Evolution des Maladies Infectieuses, IRD/CNRS/UM1 (UMR 2724), Montpellier, France; 2 IRD, UMR 177 IRD-CIRAD, Centre International de Recherche-Développement sur l'Elevage en zone Subhumide (CIRDES), Bobo-Dioulasso, Burkina-Faso; 3 CNRS, Délégation Languedoc-Roussillon, Montpellier, France; The Fox Chase Cancer Center, United States of America

## Abstract

Leishmaniases remain a major public health problem today (350 million people at risk, 12 million infected, and 2 million new infections per year). Despite the considerable progress in cellular and molecular biology and in evolutionary genetics since 1990, the debate on the population structure and reproductive mode of *Leishmania* is far from being settled and therefore deserves further investigation. Two major hypotheses coexist: clonality versus sexuality. However, because of the lack of clear evidence (experimental or biological confirmation) of sexuality in *Leishmania* parasites, until today it has been suggested and even accepted that *Leishmania* species were mainly clonal with infrequent genetic recombination (see [1] for review). Two recent publications, one on *Leishmania major* (an in vitro experimental study) and one on *Leishmania braziliensis* (a population genetics analysis), once again have challenged the hypothesis of clonal reproduction. Indeed, the first study experimentally evidenced genetic recombination and proposed that *Leishmania* parasites are capable of having a sexual cycle consistent with meiotic processes inside the insect vector. The second investigation, based on population genetics studies, showed strong homozygosities, an observation that is incompatible with a predominantly clonal mode of reproduction at an ecological time scale (∼20–500 generations). These studies highlight the need to advance the knowledge of *Leishmania* biology. In this paper, we first review the reasons stimulating the continued debate and then detail the next essential steps to be taken to clarify the *Leishmania* reproduction model. Finally, we widen the discussion to other Trypanosomatidae and show that the progress in *Leishmania* biology can improve our knowledge of the evolutionary genetics of American and African trypanosomes.

## Introduction

A major challenge with parasitic pathogens is determining disease epidemiologies and transmission cycles. In addition to the different biotic and abiotic factors involved in the infectious pattern, the pathogen's reproductive system is one of the basic biological processes that condition the microorganism's ecology and disease spread. It has been largely demonstrated that improved knowledge on the population structure and reproductive strategy of pathogens provides key information to gaining a better understanding of transmission patterns and indispensable information for diagnostic and epidemiological inquiries as well as drug and vaccine development [2]–[5]. Nevertheless, it is often difficult to obtain such information on these microorganisms because the experimental investigations are often difficult to conduct and sometimes even almost impossible to develop. For a majority of pathogens, including the Trypanosomatidae family, the reproductive strategy was mainly deduced from population genetics analysis. The main goal of this review is to detail the progress made in the knowledge of the *Leishmania* reproduction model over the past 20 years.

**Figure 1:**
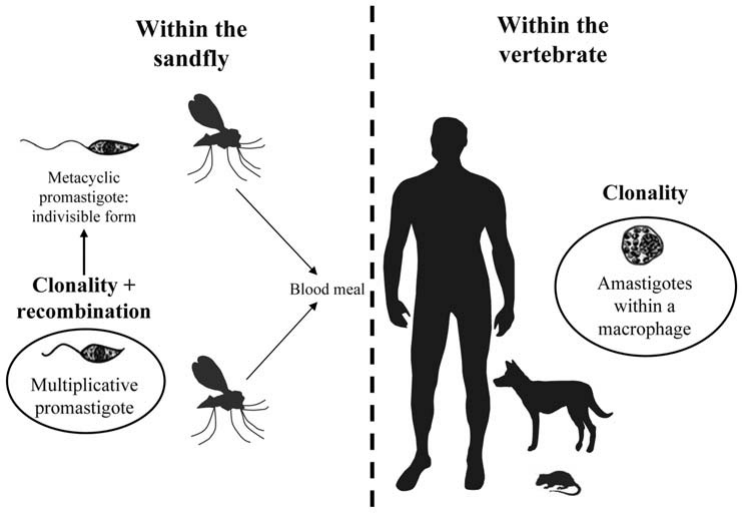
Schematic life cycle of *Leishmania* parasites. The life cycle starts when a parasitized female sandfly takes a blood meal from a vertebrate host (e.g., a human). As the sandfly feeds, infectious promastigote (metacyclic) forms of the parasite enter the vertebrate host. Within the vertebrate host, these forms are phagocytosed by macrophages where they differentiate into amastigote forms. The life cycle is completed when, during a blood meal, a female sandfly ingests infected macrophages. The parasites transform into multiplicative promastigotes inside the sandfly, and after migration into the sandfly's proboscis, promastigotes transform into metacyclic promastigotes (infectious form) and must be delivered to a new host for the life cycle to continue. The possible locations of clonality in the two hosts and of sexual events (recombination between two individuals) in the vector are indicated (figure adapted from http://www.dpd.cdc.gov/dpdx).

Protozoan parasites of the *Leishmania* genus are responsible for human leishmaniases, a serious public health problem. Indeed, leishmaniases are worldwide vector-borne diseases in humans and domestic animals with approximately 350 million persons at risk and 2,357,000 new cases per year [6]. These parasitoses occur on all continents except Antarctica. *Leishmania* parasites have a complex life cycle (Figure 1). They are present in extremely diverse ecosystems and are able to infect a wide variety of mammals. In humans, the majority of *Leishmania* infections lead to asymptomatic cases. However, when the disease is declared, it is expressed in a variety of more or less serious clinical forms: cutaneous, mucocutaneous, and visceral. For all these reasons, *Leishmania* provides a complex biological model from the ecological, genetics, and phylogenetic points of view [1]. Despite numerous studies since the 1990s and recent advances in the molecular genetics of these organisms, the *Leishmania* parasite's mode of reproduction is still under debate. Two major hypotheses are postulated: clonality versus sexuality. At this stage, it seems useful to review and clarify the arguments that fuel this debate and discuss the latest hypotheses. Finally, we will detail the next essential steps needed to advance our understanding of the reproduction mode of *Leishmania* and the usefulness of these approaches for other kinetoplastid parasites such as American and African trypanosomes.

## Mode of Reproduction in the *Leishmania* Genus: The Debate

In the *Leishmania* genus, asexual reproduction was proposed long ago as the main mechanism of reproduction. Since 1990, Tibayrenc et al. have proposed the clonal theory for all or most *Leishmania* species [5], [7]–[9]. This theory was principally based on the concept that offspring are genetically identical to their parents. Consequently, several criteria have been proposed to test this theory (e.g., fixed heterozygosity, absence of recombinant genotypes, deviation from Hardy-Weinberg expectations, widespread identical genotypes, linkage disequilibrium, and a correlation between two independent sets of genetic markers (see glossary in Box 1)) [9]. Nevertheless, based on several observations of the interspecific hybrid profiles [10]–[13], the occurrence of infrequent recombination events was not excluded for *Leishmania*. At the same time, this clonal hypothesis has been challenged by other authors. Indeed, based on molecular karyotype analysis using the pulse field gel electrophoresis (PFGE) technique, Bastien, Blaineau, and Pagès argued for a predominance of automixis (corresponding in this case to the definition of autogamy; see Box 1) [14], [15]. In this study, for each chromosome analyzed, they observed a variation in the size of subtelomeric regions (which corresponds to different chromosome forms). Moreover, the results showed that, in each individual strain, only one chromosome form was observed and that, at the population level, various recombinations were observed across these different chromosome forms. To explain these results, authors proposed the existence of a predominantly autogamic sexual reproductive mode. Similarly, the existence of genetic recombination was brought back to the debate in 2007 by Kuhls et al. [16], who proposed the possible existence of selfing (producing an excess of homozygosity; see Box 1) on the basis of multilocus microsatellite typing performed on allopatric (see Box 1) *Leishmania* samples (91 strains collected in 18 different countries). However, because of the lack of clear evidence (experimental or biological confirmation) of sexuality in *Leishmania*, it was largely accepted that *Leishmania* species were mainly clonal with infrequent genetic events (see [1] for review).

**Box 1. Glossary**

- Allopatric (antonym: sympatric): Species or populations occupying separate geographic areas.
- Aneuploidy: The occurrence of one or more extra or missing chromosomes in the same genome.
- Autogamy: A sexual reproductive mode where zygotes are produced by the fusion of two gametes that were produced by the same parental individual. This results in an increased homozygosity over time.
- Automixis: A parthenogenetic reproductive mode in which the (unfertilized) eggs are diploid, although meiosis occurs during oocyte formation. The diploidy of the egg is either restored a posteriori or is due to a genome duplication of the female gamete mother cell just before meiosis (see [22] for a more detailed explanation).
- Clonality: Reproduction with no sex. The descent is identical to the parental individual. Theoretically, excess heterozygotes and linkage disequilibrium are expected.
- Endogamy: Recombination between related individuals, e.g., selfing (see below).
- Inbreeding: A measure of the probability of identity by descent that tends to increase as a result of closed mating systems (selfing [see below], sib-mating) or limited population sizes.
- Linkage disequilibrium: A characteristic expressing the nonrandom association between different loci (generally by pair). Many different factors (population structure, closed mating system, selection, etc.) can generate and maintain statistical association between loci.
- Selfing: A sexual reproductive mode where an individual self-fertilizes its own ovules with its own spermatozoids. The resulting progenies tend to become highly homozygous at most genetic loci.
- Wahlund effect: A phenomenon that occurs when a sample consists of individuals that were sampled from genetically differentiated subpopulations. This produces a loss of heterozygosity compared to expected heterozygosity under the assumption of the random union of gametes.

These previous studies did not always make use of suitable or sufficient molecular tools, sampling, or data interpretations to reach a definitive conclusion on the reproduction mode of *Leishmania* parasites. First, the genetic markers used (such as multilocus enzyme electrophoresis [MLEE], random amplified polymorphic DNA [RAPD], PFGE, and restriction fragment length polymorphism [RFLP]) have inherent limitations that make inferences on the population's genetic structure unreliable [17]. Indeed, these molecular markers have low resolution (MLEE or RFLP) or are dominant (RAPD) or multifactorial (reflecting global genomic organization in PFGE). Second, samplings were often too limited for robust population genetic interpretations because of small sample sizes and wide geographical and/or temporal distributions, which have important consequences on the structure of the data. Indeed, living organisms generally are not homogeneously distributed across their environment, and therefore most natural populations are subdivided into more or less small subpopulations. Natural populations are submitted to genetic drift and thus likely to display variable allele frequencies over time. This substructuring of populations has a great influence on the distribution of genetic information in terms of allelic frequencies. Theoretically, if allelic frequencies are not identical between subpopulations, an apparent loss of heterozygotes can be expected when considering the entire population compared to Hardy-Weinberg expectations; this is the Wahlund effect [18] (see Box 1). Third, clonality was mainly inferred from analyses of linkage disequilibria observed across loci. However, computer simulations showed that linkage disequilibria do not provide reliable measurements of the proportion of clonal or sexual reproduction in a population [19]. Furthermore, it was recently demonstrated that Wahlund effects (inappropriate sampling; see Box 1) in clonal populations can lead to highly biased perceptions of linkage disequilibrium [19]–[21].

Theoretical studies showed that strong heterozygote excesses are expected in diploid clones [22]–[26]. Judson and Normak reported that, for ancient diploid asexual lineages, the two alleles at one locus within an individual are expected to be highly divergent since they will accumulate different mutations independently [27], which results in substantial intralocus divergence. This phenomenon, called the Meselson effect (after the name of the leader of the laboratory in which this phenomenon was first described), has been empirically documented in bdelloid rotifers [23], [24]. Moreover, it is important to specify that even at an ecological time scale, clonal reproduction is expected to produce high heterozygosities. Thus, the homozygosity excesses recurrently observed in *Leishmania* population genetics studies contradict a dominant and ancient clonal reproductive mode.

## More Sex in *Leishmania*?

Nowadays, with the development of elaborate experimental techniques and sophisticated statistical tools, our understanding of the evolutionary processes that govern the propagation of these parasites is continuously improving. Recently, two papers once again challenged the clonal hypothesis on the basis of empirical and experimental data. On one hand, a publication reporting a population genetics study on 125 human strains of *Leishmania* (*Viannia*) *braziliensis* based on 12 microsatellite loci revealed very high levels of homozygosity within samples [17]. The population structure studied at a finer scale by a Bayesian analysis demonstrated that a large part of this was explained by population substructuring (Wahlund effect). Nevertheless, there was still a substantial heterozygote deficit, which is theoretically incompatible with a predominant clonal mode of reproduction at ecological time scales, as explained above. Indeed, the high homozygosity obtained was more in agreement with the occurrence of mating between individuals from related strains (endogamy; see Box 1). We then proposed that *L*. (*V*.) *braziliensis* parasites could alternate between different modes of reproduction: clonality in both vertebrate host and insect vector and endogamy within the insect vector (as has been shown to occur for other kinetoplastid parasites, such as *Trypanosoma brucei* s.l. [28]), with occurrence of quite frequent recombination events between different (i.e., genetically divergent) individuals (see Figure 1) [29]. On the other hand, in parallel, the occurrence of mating in *Leishmania* parasites has been validated by a recent experimental study [30]. The authors provided evidence of genetic exchanges (i.e., recombination between individuals from two strains of the same species) within the vector host of *Leishmania major*. Using transgenic *Leishmania* strains resistant to different selective drugs, Akopyants et al. infected natural sandflies and then isolated parasites resistant to both drugs. They succeeded in producing hybrid progenies, characterized by full genomic complements from recombination between two related individuals, but with kinetoplast DNA maxicircles from only one parent. It should be noted that seven out of 18 hybrid progeny clones studied showed triploid DNA content, which could be explained by the capacity of *Leishmania* parasites to cope with aneuploidy (see Box 1). The authors proposed that recombination occurs only in the vector and that hybrid progeny are transmitted to the mammalian vertebrate host by sandfly bites. For the first time, this study directly and experimentally proved the existence of mating in *L. major*. These two studies combined challenge the assumption that the *Leishmania* parasites' mode of reproduction is overwhelmingly clonal.

## What Will Be the Next Steps?

At this stage, it appears essential to extend population genetics approaches to different *Leishmania* species and various environments. Analysis of genetic variation at different hierarchical levels is often the only way to investigate natural population characteristics such as gene flow and reproductive strategies [31]. Moreover, given the observation of high substructuring of the population observed in the empirical study described above [17], working at finer geographic scales to detect and limit the substructuring (Wahlund effect) of such pathogens would be a desirable goal for future studies [17]. Thus, exploring the genetic patterns of these parasites (allelic frequencies, heterozygosity rates, or linkage disequilibrium) at microgeographic scales would be the next challenge. This will require samples taken in small geographic areas, within short time windows, and from different host species involved in the transmission cycle (i.e., symptomatic and asymptomatic hosts and different vector species). Very few studies fulfill these conditions, often because of problems arising in the field and/or limitations in molecular techniques (i.e., lack of sensitivity for characterizing parasites in asymptomatic hosts). Some as yet unpublished results from our team (S. Kako Ouraga, V. Rougeron, and A–L. Bañuls, unpublished data.) suggest that the demographic unit could reside in the individual host itself. We developed a pioneering experiment consisting of isolating each *Leishmania* cell by cell, providing a whole genome amplification on each cell, and then amplifying the microsatellite DNA of the parasite by a nested PCR (a technique developed by the team of F. Prugnolle for *Plasmodium falciparum*; personal communication, unpublished data). The preliminary tests were made on two different human strains of *L. V. braziliensis*, CH22B and CH25B, collected in Peru in 1994 from two different patients and known to be heterozygous at several loci [17]. Single-cell genotypic characterization revealed the actual coexistence of multiple genetic entities (i.e., genetically distinct *Leishmania* individuals) within each of these strains (CH22B and CH25B). This unexpected result strongly suggests that for these parasites the most nested subpopulation unit (the smallest demographic unit) might be individual hosts, a too frequently neglected scale for infectious agents, as stressed in recent papers [21], [32]. Thus, in future studies, it would be indispensable to attempt to analyze population genetic data from the most nested subpopulation. Ignoring these scales should invariably lead to more or less high-magnitude Wahlund effects, depending on the degree of isolation between these micro-subpopulations. We believe that this type of experiment should be generalized in order to improve our knowledge of *Leishmania* reproductive strategy.

Another step to define the evolutionary mechanisms involved in mating is to characterize the sexual events occurring within the vector in detail. Volf et al. suggested applying high-resolution imaging to identify when, where, and how these phenomena occur in the sandfly gut [33]. Moreover, they explained that stressful conditions applied in the sandfly gut can induce genetic exchanges between *Leishmania* parasites. This suggests that the environment could play a key role in the frequency of mating events in *Leishmania*, justifying the need to analyze the reproduction model in diverse environments and within various species (see above).

The progress in *Leishmania* biology and the development of experimental models will improve our knowledge of the evolutionary genetics of the Trypanosomatidae family. Empirical studies are in agreement with a purely clonal mode of reproduction for *Trypanosoma brucei gambiense* type 1 [34], while other studies have experimentally proved the possibility of recombination in the tsetse vector for other *T. brucei* subspecies [35]–[43]. Nevertheless, because *Trypanosoma brucei brucei*, *T. brucei gambiense* type 2, and *Trypanosoma brucei rhodesiense* are themselves extremely heterogeneous [35] and thus probably composed of different entities, the frequency of such events remains an unanswered question. These results show that different subspecies (or species given that trypanosomes' subspecific status is questionable) can display different reproductive strategies, suggesting how important it is to explore the reproduction model within a panel of different species before any definitive conclusion can be drawn for *Leishmania*. Regarding *Trypanosoma cruzi*, the genetic profiles observed in the literature (strong linkage disequilibrium and prevalent heterozygote deficits) [9], [44]–[47] may suggest evolutionary processes similar to those found in *L. V. braziliensis*. Moreover, Gaunt et al., by producing hybrid clones, showed that *T. cruzi* has an extant capacity for genetic exchange [46]. For all these reasons, research based on experimental techniques combined with sophisticated empirical approaches must be developed considering different species and various environments in order to provide a better understanding of the reproductive mode and the transmission cycle of these protozoan parasites.

## Conclusion

In summary, we are still far from complete knowledge of the mechanisms that govern the multiplication, propagation, and diversity of *Leishmania* in particular and in kinetoplastids in general. It should be noted that we have not detailed the problem of aneuploidy here, which cannot be neglected since it has been largely demonstrated in previous investigations [14], [48] and recently confirmed experimentally [30]. Although no genetic data allow us to suspect a clear impact of aneuploidy for any of the microsatellite loci used so far in empirical approaches, this phenomenon should not be ignored in future studies. Additionally, although none of the published results on microsatellites suggests the existence of any gene conversion, the possibility of gene conversion has to be specifically studied in future population genetic studies. These parasites apparently have unsuspected resources to survive and propagate, and understanding why and how is of primary relevance for better knowledge of their epidemiology and evolutionary potential.
